# Does viral circulation in slums have a global impact? The lesson learned from SARS-CoV-2 circulation in *Complexo de favelas da Maré*, Rio de Janeiro, Brazil

**DOI:** 10.3389/fmicb.2025.1483895

**Published:** 2025-02-12

**Authors:** Monique Cristina dos Santos, Natalia Fintelman-Rodrigues, Aline de Paula Dias da Silva, Rodolfo Leandro Nascimento Silva, Victor Corrêa Seixas, Amanda A. Batista-da-Silva, Marcelo Alves Ferreira, Patrícia T. Bozza, Fernando A. Bozza, Thiago Moreno L. Souza

**Affiliations:** ^1^Laboratório de Imunofarmacologia, Instituto Oswaldo Cruz (IOC), Fundação Oswaldo Cruz (Fiocruz), Rio de Janeiro, Brazil; ^2^National Institute for Science and Technology on Innovation in Diseases of Neglected Populations (INCT/IDPN), Center for Technological Development in Health (CDTS), Fiocruz, Rio de Janeiro, Brazil; ^3^Instituto de Biologia, Universidade Federal Fluminense (UFF), Rio de Janeiro, Brazil; ^4^Instituto Nacional de Infectologia Evandro Chagas, Fiocruz, Rio de Janeiro, Brazil; ^5^Department of Industrial Engineering, Pontifical Catholic University of Rio de Janeiro, Rio de Janeiro, Brazil; ^6^D’Or Institute for Research and Education, Rio de Janeiro, Brazil; ^7^Tecgraf Institute, Pontifical Catholic University of Rio de Janeiro, Rio de Janeiro, Brazil

**Keywords:** COVID-19, SARS-CoV-2, slum, Rio de Janeiro, Brazil

## Abstract

Because of growing inequalities, more than one-third of the worldwide population is expected to live in slums by 2050. Although slum dwellers are at increased risk of infectious diseases, this population may have been overlooked with respect to the sustainability of virus evolution. In this study, we aimed to analyze the genetic diversity and evolution of SARS-CoV-2 in the *Complexo de Favelas da Maré* slum, Rio de Janeiro, Brazil, and assess its impact on the global spread of the virus. We found that this slum harbored multiple strains of SARS-CoV-2, and its amplification and genetic diversity connected with the global circulation from 2020 to 2022. Thus, enhancing surveillance in slums could be important for future epidemic/pandemic preparedness by connecting virus genetic diversity in this region with its circulation at divergent locations.

## Introduction

From the early 2019 coronavirus disease 2019 (COVID-19) pandemic outbreak to the subsequent dissemination of severe acute respiratory coronavirus 2 (SARS-CoV-2), certain scenarios may have favored global virus spread (Brainard et al., 2023). These scenarios are complex and not fully understood. Both developed and developing countries suffer from high mortality and the emergence of variants of concern (VoC) (Earnest et al., 2022; Gräf et al., 2021; Tallarita et al., 2022; Trombetta et al., 2022; Worobey, 2021), possibly because of the lack of understanding of the disease’s natural history, virus routes of transmission among asymptomatic/presymptomatic individuals, susceptibility and transmissibility of specific age groups, difficulty in implementing preventive measures (Lee et al., 2020), and the inability to reduce the chains of transmission (Brainard et al., 2023). Nevertheless, in low- and middle-income countries (LMICs), the poorest live in slums and are at greater risk of respiratory pathogens, such as *Mycobacterium tuberculosis* (Noykhovich et al., 2019), measles virus (Raoot et al., 2016), and SARS-CoV-2 (Friesen and Pelz, 2020). An in-depth analysis of SARS-CoV-2 circulation in these areas is important because accurate data collection in slums can be challenging. Informal settlements, along with a lack of access to diagnose COVID-19 and underreporting of cases (Bakibinga et al., 2019) make SARS-CoV-2 circulation in slums encrypted. Overcrowded homes make social distancing impossible (Von Seidlein et al., 2020). Poor sanitation increases the overall risk of disease transmission (Health Equity, n.d.). Limited healthcare access leads to delayed case management, resulting in increased mortality. Economic insecurity may also have caused the poorest people to avoid lockdowns to continue working (King et al., 2023).

These socioeconomic and demographic limitations, constant health vulnerabilities, and the rise of anti-science movements in a less educated population may have translated into an increased case–fatality ratio (CFR) in slums. Because of growing inequalities worldwide, one to two-thirds of the global population is predicted to live in slums by 2050 (Perlman, 2010). Thus, it is pivotal to better comprehend the dynamics of pathogen circulation in slums as part of pandemic preparedness and epidemic responses.

In the city of Rio de Janeiro, state of Rio de Janeiro, Brazil, the largest slum is *Complexo de Favelas da Maré* (in Portuguese: Tide Slum Complex; henceforth referred to as *Maré*). At *Maré*, the COVID-19 CFR reached 16%, 2- to 3-fold higher than that of the rest of the city (Batista-Da-Silva et al., 2023; Canal e Boletim, n.d.; Painéis Epidemiológicos Painel Rio COVID-19, n.d.). Because of this high mortality ratio, massive virus circulation may have occurred at *Maré*. Thus, we aimed to catalog SARS-CoV-2 genetic diversity and evolution at this slum and investigate whether the viral strains we identified remained there or whether they were linked to the global spread of the virus. In fact, *Maré* harbored multiple strains of SARS-CoV-2, and its amplification and genetic diversity connected with the global circulation from 2020 to 2022. Thus, enhancing surveillance in slums could be important for future epidemic/pandemic preparedness by connecting virus genetic diversity in this region with its circulation at divergent locations.

## Materials and methods

### Patients, samples, and RT-PCR

Nasopharyngeal swabs (NSs) were collected from residents of *Maré* under ethical approval 30650420.4.1001.0008 (by the National Research Ethics Commission, CONEP). Informed consent was obtained from all participants or patient representatives. Data anonymization was used in this study. The samples were collected during 2020 (August to October), 2021 (March to October), and 2022 (January to November), when a multidisciplinary task force was mobilized to medical assistance at *Maré*, and when the pandemic waves were peaking in Brazil (Brazil-COVID-19 Overview-Johns Hopkins, n.d.). Samples were collected from patients with fever (>37°C) and cough who presented to the task force on the basis of spontaneous demand. The task force was composed of a multidisciplinary team of health professionals called the *Dados do Bem* (in English data for good) project (Batista-Da-Silva et al., 2023; Dantas et al., 2021), which involved the Instituto D’or de Pesquisa e Ensino (IDOR) and the Instituto Nacional de Infectologia Evandro Chagas Filho (INI), Fiocruz.

NS total RNA was extracted with a QIAamp Viral RNA Kit (Qiagen, Hilden, Germany). RT-PCR was performed according to our previous publication (Fintelman-Rodrigues et al., 2021).

### SARS-CoV-2 genome assembly, bioinformatic processing, and assignment of variants

SARS-CoV-2 complete genomes were obtained via ATOPlex SARS-CoV-2 Panel v2.0 (MGI Tech Co., Shenzhen, China), as previously described (Fintelman-Rodrigues et al., 2021). RNA from samples with Ct ≤ 25 was reverse-transcribed into cDNA and amplified for targeted enrichment and dual indexing. Using samples with Ct ≤ 25 indicates that we selected samples with high viral loads to facilitate sequencing. DNA nanoballs were amplified by rolling circles and paired-end sequenced (150 nt) on the MGISEQ-g400 platform (MGI Tech Co., Shenzhen, China).

The raw genomic data were processed via the UseGalaxy platform^1^ with a SARS-CoV-2 reference sequence (Wuhan-hu-1 isolate, GenBank MN908947.3) and a BED file containing primer coordinates from the MGI ATOPlex panel.^2^ FASTQ files were preprocessed by FASTP v.0.20.1 to remove adapters and reads shorter than 50 bp (−l 50). Reference mapping and genome assembly were carried out with BWA-MEM v. 0.7.17 in default mode. BAM files were filtered by quality (−q 20) to exclude (−F) unmapped reads (and their mate pairs) and non-primary alignments (SAMtools view v.1.13). The primer binding sites were trimmed (iVar trim v.1.3.1; −m 1 −q 0 −s 4 −e), and the file was aligned to the reference genome with LoFreq v.2.1.5 (with indel filtering by the Dindel algorithm). Variants were called with iVar variants v.1.3.1 (−q 30 −t 0.51 –pass_only), and output VCF files were used to call consensus with BCFtools v.1.10. SARS-CoV-2 consensus genomes were aligned and assigned to global outbreak lineage next-strain clades with NextClade v.1.5.1.^3^ The latter also provided quality check reports for each consensus sequence and generated an output JSON file for subsequent phylogenetic assessment in Auspice v.0.8.0.^4^

### Haplotypic analysis

Neutrality tests and haplotype data were generated with DnaSP v6.12.03. The haplotype network was inferred with TCS networks (Clement et al., 2000) and drawn with PopART v1.7. The haplotypic (h) and nucleotide (*π*) diversities were calculated via the DNAsp application v.6. The calculations of F’s for Fu and D for Tajima were performed by Arlequin v.3.5.2.2 within the R program v.4.0.3. Finally, the modeling of the haplotype network was performed in PopArt v.1.7 through the TCS network with statistical parsimony and a confidence level of 95%.

## Results

### Sociodemographic characteristics of the patients

In total, we diagnosed and sequenced NS samples from 501 patients (GenBank Accession codes #OR538895–OR539182 and PQ567385–PQ567517, PQ567519, and PQ577902–PQ577980) from 2020, 2021, and 2022 from *Maré* residents [53% female and aged 37 ± 24 years (median ± SD)]. Although patients are recruited on the basis of spontaneous demand, their ages and ethnic backgrounds are similar to those of dwellers from *Maré*, who were censored in 2022 (Batista-Da-Silva et al., 2023; Canal e Boletim, n.d.; IBGE | Biblioteca, n.d.) (Supplementary Figure S1).

### Early SARS-CoV-2 circulation at *Maré*

**Figure 1:**
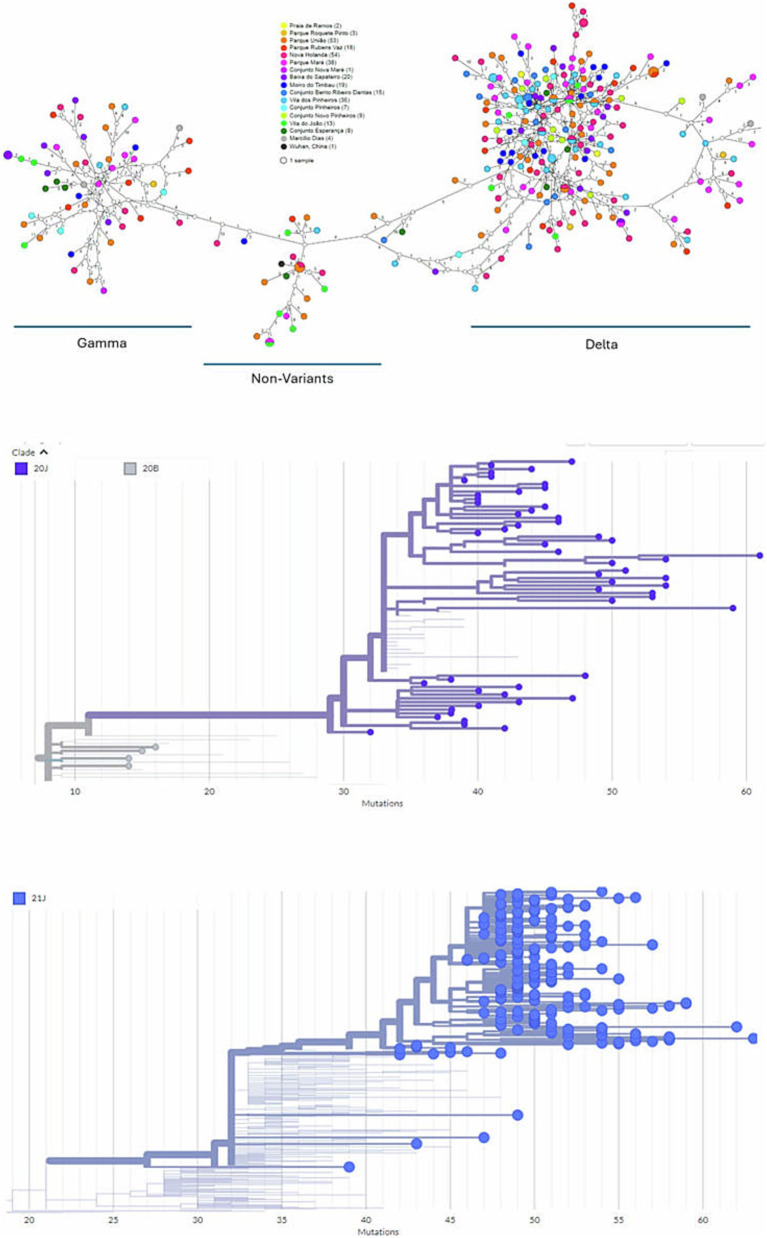
Positive nasopharyngeal swabs from dwellers of *Complexo de Favelas da Maré (Maré)*, Rio de Janeiro, Brazil, from 2020 to 2022 were sequenced (Atoplex version 2.0, using the pair-end of 150 nucleotides in the DNBSEQ-G50 sequencer; MGI Tech, https://en.mgi-tech.com/). Full-length consensus genomes were generated with quality scores >30 and depths >10. **(A)** The color represents the place of residence among *Maré* sublocations and was analyzed in a haplotype network inferred via the TCS method (statistical parsimony). For phylogenetic analysis, the maximum-likelihood method was used with consensus sequences and publicly available datasets from NextClade v.1.5.1. Diagrams of mutations across the genomes of the emphasizing strains 20J **(B)** and 21J **(C)** are presented. The original sequences are marked as spheres (GenBank Accession codes #OR538895–OR539182).

During the 2020–2021 COVID-19 pandemic, *Maré* had a CFR of 10–16% (Batista-Da-Silva et al., 2023; Canal e Boletim, n.d.), which is twice as high as the CFR in the city of Rio de Janeiro (Painéis Epidemiológicos Painel Rio COVID-19, n.d.). Because of the high mortality ratio at *Maré*, we were interested in better comprehending the viral dynamics in this slum region. With respect to this period of early SARS-CoV-2 introduction, prior to vaccination campaigns, we looked for chains of transmission within the community and built a haplotype network for SARS-CoV-2 genomes. Haplotypes that clustered independently of the residence of the dweller within 5 km^2^ were called *Maré* (Figure 1). In fact, the SARS-CoV-2 haplotypes were distributed according to the variant of concern to which they belong (VoC) (Figure 1A). We identified 265 haplotypes, with 6 shared pairs and 4 detected in the same location (Figure 1A), suggesting some level of intraslum transmission. Among these strains at *Maré*, we also found high haplotype and nucleotide diversity indices, along with significant Fu and Li parameters (Table 1), indicating that new SARS-CoV-2 genomes entered the community and evolved there, which is consistent with massive virus introduction and circulation in this setting.

**Table 1:** SARS-CoV-2 diversity at *Complexo de Favela da Maré.*

Variants	*N*	h	S	Hd	π	k	Tajima’s D	Fu and Li’s
20A	13	14	73	1.000	0.00069	19.319	−0.70222	−0.90178
20B	15	15	81	0.992	0.00044	12.950	−2.01663*	−2.82136*
20 J	45	52	201	0.999	0.00050	13.413	−2.51051*	−4.99782*
21 J	215	207	365	0.999	0.00035	9.712	−2.68439*	−9.39252*
22B	213	192	29,459	0,998	0,37,881	11.298651	−0.17862	36.651
Total	501	452	887	0,999	0,00145	38.501	−2.16354*	−12.26969*

*N* = number of individuals; h = number of haplotypes; S = number of polymorphic sites; Hd = haplotype diversity (standard deviation); π = nucleotide diversity (standard deviation); k = average number of nucleotide differences. *Indicates a significant difference.

We also performed phylogenetic analysis to compare the SARS-CoV-2 genomes from *Maré* with those found elsewhere. Both branches of the Gamma (clade 20 J) and Delta (clade 21 J) VoCs indicate that SARS-CoV-2 genomes from *Maré* not only root additional sequences of this virus in the slum region but are also ancestral to pandemic coronavirus genomes from other countries (Figures 1B,C; Supplementary Tables S1, S2). SARS-CoV-2 gamma VoC from *Favela da Maré* is associated with cases in Brazil, the USA, and Switzerland (Supplementary Table S1). Delta VoC from the Brazilian slum region is associated with other cases from this country, the USA, India, South Africa, China, and Switzerland (Supplementary Table S2). Delta samples from *Maré* early presented the spike mutations D614G, P681R, and L452R, which are associated with increased infectivity and immune escape. The discovery of SARS-CoV-2 genomes with more competitive features from *Maré* is also supported by a tendency toward directional selection depicted by Tajima’s D parameter (Table 1).

### Omicron circulation at *Maré*

**Figure 2:**
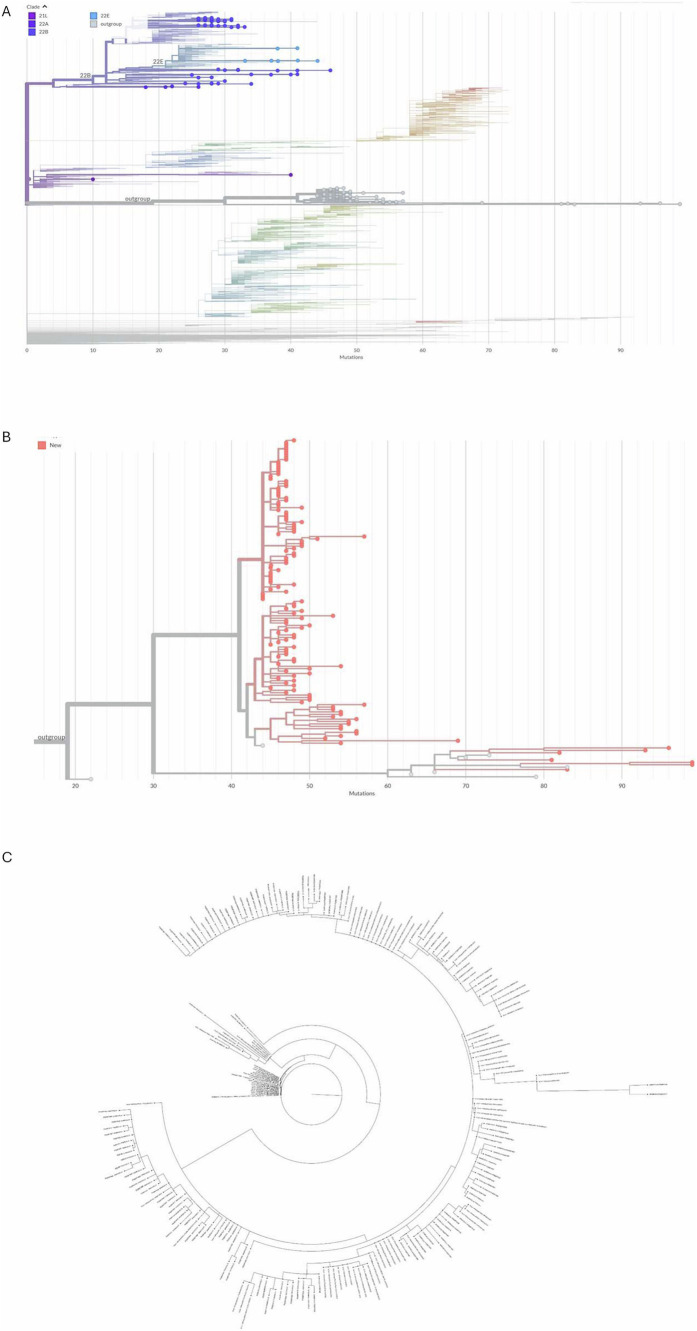
Positive nasopharyngeal swabs from dwellers of *Complexo de Favelas da Maré (Maré)*, Rio de Janeiro, Brazil, from 2022 were sequenced (Atoplex version 2.0, using the pair-end of 150 nucleotides in the DNBSEQ-G50 sequencer; MGI Tech, https://en.mgi-tech.com/). Full-length consensus genomes were generated with quality scores >30 and depths >10. For phylogenetic analysis, the maximum-likelihood method was used with consensus sequences and publicly available datasets from NextClade v.1.5.1 highlighting the strains 21L, 22A, 22B, 22E, and an out-group **(A)**. A phylogeny of mutations across the genomes of the emphasizing strains in the new omicron out-group (in red) compared with predecessor strains from this same out-group (in gray) is displayed **(B)**. Further phylogenetic analysis was performed with samples from the out-group found in Brazil and other territories **(C)**. The original sequences are marked as spheres (GenBank Accession codes #Q567385–PQ567517, PQ567519, and PQ577902–PQ577980).

During 2022, omicron VoC took over previous strains at *Maré* (Figure 2). Indeed, the SARS-CoV-2 omicron strains 21 L, 22A, 22B, and 22E and a new out-group were observed at *Maré* (Figure 2A). SARS-CoV-2 is constantly mutating, and the emergence of a new out-group means that the virus has accumulated enough mutations to make it genetically different from the dominant circulating variants. The accumulation of genetic evidence is necessary for an out-group to be defined as a new omicron subvariant. In this sense, the study of SARS-CoV-2 evolution in the poor slum of *Maré* adds new sequences to a SARS-CoV-2 out-group derived from the BA.2 subvariant of Omicron, which was previously underrepresented (Figure 2B). This new BA.2 out-group is similar to sequences observed in all continents; sequences from *Maré* are initially rooted in a virus strain from Wales, and next, other strains from *Maré* are ancestral to cases in Brazil and Latin America (Figure 2C).

## Discussion

### The urgent need for research in underserved communities

With increasing global inequalities, more than one-third of the world’s population is projected to reside in slums by 2050 (Perlman, 2010). These communities, characterized by high population density, poverty, and inadequate sanitation, create environments where infectious diseases can easily spread (Neiderud, 2015). Studying disease transmission in these settings is vital because these areas often have a higher prevalence of infectious diseases than other urban areas do.

Understanding the dynamics of infectious agents in slums is crucial for public health interventions. However, these poor communities may have been overlooked in studies concerning the sustainability of virus evolution. Research focusing on slums such as the *Complexo de Favelas da Maré* in Rio de Janeiro, Brazil, reveals crucial insights into the genetic diversity and evolution of viruses such as SARS-CoV-2. These findings indicate that slums harbor multiple introductions of the virus, contributing to its amplification and genetic diversity and connecting with global circulation patterns.

### The *Maré* slum: a case study

In Brazil, approximately 8% of the population lives in slums (IBGE | Biblioteca, n.d.). Like other territories around the world, many Brazilian slums are in the central urban areas of cosmopolitan cities and megacities, leading to the hypothesis that a pathogen spread in these areas could reach wider dissemination. *Maré* is located just by Guanabara Bay (the 2nd largest bay in Brazil and an area of intense naval activity), is 7 km away from Rio’s International Airport, and is crossed by three of the most used highways in the city. More than 140,000 residents of this slum are distributed in more than 43,000 homes in 16 sublocations encompassing a total area of 5 km^2^, an area ranked as one of the last neighborhoods among Rio de Janeiro’s districts according to the Human Development Index (Rio em Síntese, n.d.).

In the early SARS-CoV-2 circulation at *Maré*, a diverse array of SARS-CoV-2 strains, including multiple variants of concern (VOCs), were found. This diversity suggests that the slum may have acted as an environmental reservoir for the virus, facilitating the evolution of new strains. This is represented by massive virus circulation/replication at *Maré* from 2020 to 2021, which is related to epidemics in distinct areas of the world: the USA, Switzerland, India, South Africa, and China.

By the end of 2021 and the beginning of 2022, the Brazilian population had broader immunity as the result of natural exposure and vaccination campaigns, which led to the decline of the Brazilian CFR in 2022 (Boletins Epidemiológicos COVID-19, n.d.). Nevertheless, several important features of the molecular epidemiology of SARS-CoV-2 were observed at *Maré*. During 2022, Omicron VoC circulated, and at *Maré*, we found a new out-group of genotype BA.2, which was similar to sequences observed worldwide. The SARSC-CoV-2 sequences from *Maré* subsequently became ancestral to the virus strains found in Brazil, Latin American countries, and Wales. These findings from all the years analyzed suggest that *Maré* may have played a role in the global dissemination of the virus.

The dynamic environment of slums, with overcrowding and limited resources, makes it difficult to capture a comprehensive picture of viral diversity and inform effective public health interventions. By analyzing viral genetic diversity in *Maré* via high viral load samples, we can potentially over represent dominant strains and miss less prevalent variants. Moreover, genomic tools and diversity metrics can introduce biases due to assumptions of neutral evolution. Thus, it is plausible that more longitudinal sampling strategies and universal sequencing result in greater genetic diversity.

### The urgent need for action

The data from 2022 from *Maré* on the circulation of a new Omicron out-group reinforce that the pandemic’s impact on slums underscores the urgent need for several actions. Improved infrastructure and basic services, such as clean water, sanitation, and healthcare in slums, are crucial for improving public health and resilience to future crises. Social protection programs are needed because stronger social safety nets are needed to protect vulnerable populations from economic shocks and ensure access to essential needs. Community empowerment is important for supporting community-led initiatives, and organizations can strengthen local responses to crises and promote long-term development. Therefore, enhanced surveillance in slums is critical for future epidemic and pandemic preparedness as it links viral genetic diversity in these regions with its circulation in diverse locations.

### Comparative interventions with other slums

Studies conducted in India, specifically in Mumbai, have revealed that crowding plays a significant role in the transmission of COVID-19. Research has shown that the seroprevalence in slum areas is considerably greater than that in non-slum areas, highlighting the impact of close proximity on virus spread (Malani et al., 2021). In response, experts have suggested implementing the “4 Ts” approach—tracing, tracking, testing, and treating—which is effective in interrupting the chain of transmission in Dharavi (Kaushal and Mahajan, 2021). Similarly, a multicomponent intervention in *Maré*, Brazil, incorporating community engagement, mobile surveillance, mass testing, and telemedicine, resulted in a substantial decrease in the COVID-19 fatality rate (Batista-Da-Silva et al., 2023). However, genomic analysis in *Maré* indicated that most infections originated outside the community, emphasizing the influence of resident mobility and external exposure on virus circulation. This finding underscores the need for urban planning strategies that account for human movement to effectively mitigate transmission in densely populated urban slums.

Bangladesh, with its high population density and a significant portion of its urban population residing in slums with inadequate living conditions, faces challenges in controlling the spread of COVID-19. Strict lockdown measures were difficult to maintain, resulting in the introduction of various virus clades from different parts of the world, including Europe, the United States, the United Kingdom, and Australia (Islam et al., 2021). While genomic studies have tracked the evolution of the virus in Bangladesh, further research is needed to understand how individual-level genomic changes influence the overall dynamics of transmission at the population level. The study in *Maré* provides valuable genomic data from slums over time, which can help assess viral dynamics and inform strategies for addressing future pandemics.

Comparing *Maré* to slums in India and Bangladesh reveals important differences in healthcare systems, population densities, and mobility patterns (Osuh et al., 2024). While Brazil has a universal healthcare system, access to and quality of care remain challenges. India and Bangladesh have more fragmented systems with greater reliance on private providers and non-governmental organizations. Slums in India and Bangladesh often have significantly higher population densities than those in Brazil do, posing greater challenges for public health interventions. Mobility restrictions, while present in all settings, may be more difficult to enforce in Brazilian slums because of safety concerns and geographical barriers. Indian and Bangladeshi slums experience high levels of internal migration, which can create challenges in accessing healthcare and social services.

To improve the resilience of slums to future health crises, it is crucial to invest in improved infrastructure and basic services such as clean water, sanitation, and healthcare. Community health worker programs have also been shown to be effective in increasing access to healthcare and health education in slums (Javanparast et al., 2018). By addressing these multifaceted challenges and investing in comprehensive interventions, we can better protect vulnerable slum communities from the devastating impacts of pandemics.

### Future directions

We endorse robust surveillance systems in underserved communities to track the emergence and spread of pathogens. This includes genomic sequencing, epidemiological monitoring, community-based participatory surveillance, and *a priori* epidemiological design. We also recognize that countries may face distinct challenges and often explore tailored interventions; that is, public health interventions developed in light of slum specificities may be necessary. This may involve culturally appropriate health education campaigns, community health worker programs, and improved access to healthcare services. Global public health agencies could reinforce their initiatives toward underserved communities and establish a tone for politicians around the world.

## Conclusion

The population at *Complexo de Favelas da Maré* in Rio de Janeiro, Brazil, is not only at higher risk for SARS-CoV-2, as documented by multiple virus introductions within this slum, but also the virus that circulated there evolved and correlated with epidemics in other countries/continents. Considering that a substantial part of the world population will live in slums in the next few decades, we reinforce the necessity of prioritizing these areas for health initiatives and sanitation and further advocate that slums should be prioritized to receive funds and public health efforts in epidemic/pandemic periods.

## Data Availability

The datasets presented in this study can be found in online repositories. The names of the repository/repositories and accession number(s) can be found below: https://www.ncbi.nlm.nih.gov/genbank/, OR538895–OR539182; https://www.ncbi.nlm.nih.gov/genbank/, PQ567385–PQ567517; https://www.ncbi.nlm.nih.gov/genbank/, PQ567519; https://www.ncbi.nlm.nih.gov/genbank/, PQ577902–PQ577980.

## Footnotes

^1^ www.usegalaxy.org

^2^ https://pan.genomics.cn/ucdisk/s/ERnMVz

^3^ https://clades.nextstrain.org/

^4^ https://auspice.us/
