# Supplementary material for: Does viral circulation in slums have a global impact? The lesson learned from SARS-CoV-2 circulation in Complexo de favelas da Maré, Rio de Janeiro, Brazil
Source: Front Microbiol. 2025 Feb 12;16:1483895. doi: 10.3389/fmicb.2025.1483895 (PMC11861056; doi:10.3389/fmicb.2025.1483895)
Supplement: Supplementary file 1 [file Data_Sheet_1.pdf]

Does the viral circulation in slums have a global impact? The lesson learned from early SARS-CoV-2 circulation in *Complexo de Favelas da Maré,* Rio de Janeiro, Brazil

Monique Cristina dos Santos[1,2], Natalia Fintelman-Rodrigues[1,2], Aline de Paula Dias da Silva[1,2], Rodolfo Leandro Nascimento Silva[3], Victor Corrêa Seixas[3], Amanda A. Batista da Silva[4,5], Marcelo Alves Ferreira[2], Patrícia T. Bozza[1], Fernando A. Bozza[3,6,7,*], Thiago Moreno L. Souza[1,2,*]

1- Laboratório de Imunofarmacologia, Instituto Oswaldo Cruz (IOC), Fundação Oswaldo Cruz (Fiocruz), Rio de Janeiro, Rio de Janeiro, Brazil
2-National Institute for Science and Technology on Innovation in Diseases of Neglected Populations (INCT/IDPN), Center for Technological Development in Health (CDTS), Fiocruz, Rio de Janeiro, Rio de Janeiro, Brazil
3- Instituto de Biologia, Universidade Federal Fluminense (UFF), Niterói, Rio de Janeiro, Brazil
4- Instituto Nacional de Infectologia Evandro Chagas, Fiocruz, Rio de Janeiro, Rio de Janeiro, Brazil;
5-Department of Industrial Engineering, Pontifical Catholic University of Rio de Janeiro, Rio de Janeiro, Brazil
6-D'Or Institute for Research and Education, Rio de Janeiro, Brazil;
7-Tecgraf Institute, Pontifical Catholic University of Rio de Janeiro, Rio de Janeiro, Brazil
*E-mail: thiago.moreno@fiocruz.br , fernando.bozza@fiocruz.br

SUPPLEMENTARY FIGURE

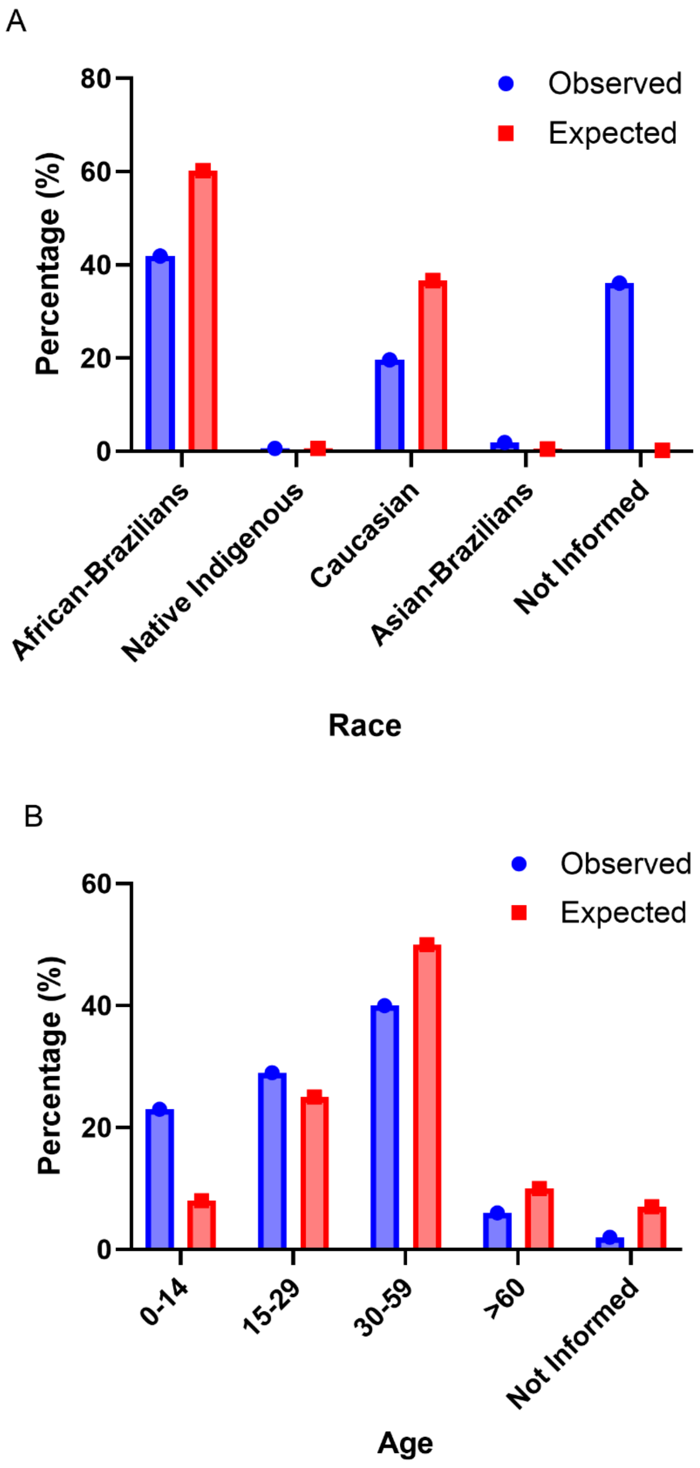

Figure 1. Descriptive comparison of the collected patients (observed) and censored individuals (expected) at *Maré*. Panel A represents the races and panel B the ages.

SUPPLEMENTARY TABLES

Supplementary Table 1 – SARS-CoV-2 genomes from Gamma VoC similar to those of strains circulating in *Complexo de Favela da Maré*

Description	*Total Score*	*Query Cover*	*E value*	*Acc. Len*	*Accession*
Severe acute respiratory syndrome coronavirus 2 isolate SARS-CoV-2/human/BRA/HCOR_A823000661/2021	*54215*	*54215*	*100%*	*99.94*	*29903*
Severe acute respiratory syndrome coronavirus 2 isolate SARS-CoV-2/human/BRA/HCOR_666488/2021	*54143*	*54143*	*100%*	*99.90*	*29903*
Severe acute respiratory syndrome coronavirus 2 isolate SARS-CoV-2/human/BRA/RJ-NVBS648GENOV827559572969/2021	*54143*	*54143*	*100%*	*99.90*	*29831*
Severe acute respiratory syndrome coronavirus 2 isolate SARS-CoV-2/human/BRA/1061/2021	*54143*	*54143*	*100%*	*99.90*	*29903*
Severe acute respiratory syndrome coronavirus 2 isolate SARS-CoV-2/human/USA/VSP2639/2021	*54141*	*54141*	*100%*	*99.89*	*29907*
Severe acute respiratory syndrome coronavirus 2 isolate SARS-CoV-	*54137*	*54137*	*100%*	*99.89*	*29866*

2/human/BRA/CD1739-P4/2020					
Severe acute respiratory syndrome coronavirus 2 isolate Switzerland/GE-33242357/2021 genome assembly	*54135*	*54135*	*100%*	*99.89*	*29865*
Severe acute respiratory syndrome coronavirus 2 isolate Switzerland/GE-33173470/2021 genome assembly	*54135*	*54135*	*100%*	*99.89*	*29850*
Severe acute respiratory syndrome coronavirus 2 isolate Switzerland/GE-33173437/2021 genome assembly	*54135*	*54135*	*100%*	*99.89*	*29863*
Severe acute respiratory syndrome coronavirus 2 isolate Switzerland/GE-33154798/2021 genome assembly	*54135*	*54135*	*100%*	*99.89*	*29865*
Severe acute respiratory syndrome coronavirus 2 isolate SARS-CoV-2/human/USA/FAA4B8136EQ/2021	*54131*	*54131*	*100%*	*99.89*	*29821*
Severe acute respiratory syndrome coronavirus 2 isolate SARS-CoV-2/human/USA/FA6C70F0FCE/2021	*54131*	*54131*	*100%*	*99.89*	*29806*

Supplementary Table 2 – SARS-CoV-2 genomes from Delta VoC strains similar to those of strains circulating in *Complexo de Favela da Maré*

Description	*Total Score*	*Query Cover*	*E value*	*Acc. Len*	*Accession*
Severe acute respiratory syndrome coronavirus 2 isolate SARS-CoV-2/human/IND/SRHU_58/2021	*54200*	*54200*	*100%*	*99.93*	*29903*
Severe acute respiratory syndrome coronavirus 2 isolate SARS-CoV-2/human/USA/CA-SEARCH-49941/2021	*54194*	*54194*	*100%*	*99.92*	*29845*

Severe acute respiratory syndrome coronavirus 2 isolate SARS-CoV-2/human/France/10029DT/2020	*54194*	*54194*	*100%*	*99.92*	*29903*
Severe acute respiratory syndrome coronavirus 2 isolate SARS-CoV-2/human/USA/NY-PRL-210217_00L24/2021	*54189*	*54189*	*100%*	*99.92*	*29752*
Severe acute respiratory syndrome coronavirus 2 isolate SARS-CoV-2/human/ZAF/NHLS-UCT-GP-5276/2020	*54189*	*54189*	*100%*	*99.92*	*29823*
Severe acute respiratory syndrome coronavirus 2 isolate SARS-CoV-2/human/ZAF/NHLS-UCT-GP-5326/2020	*54189*	*54189*	*100%*	*99.92*	*29820*
Severe acute respiratory syndrome coronavirus 2 isolate SARS-CoV-2/human/ZAF/NHLS-UCT-GS-0087/2020	*54189*	*54189*	*100%*	*99.92*	*29820*
Severe acute respiratory syndrome coronavirus 2 isolate SARS-CoV-2/human/CHN/SH-P94-B-3-Europe-UK/2020	*54189*	*54189*	*100%*	*99.92*	*29689*
Severe acute respiratory syndrome coronavirus 2 isolate SARS-CoV-2/human/CHN/SH-P125-3-Asia-UAE/2020	*54189*	*54189*	*100%*	*99.92*	*29689*
Severe acute respiratory syndrome coronavirus 2 isolate SARS-CoV-2/human/SWE/14_SE300_DE20-005545/2020	*54189*	*54189*	*100%*	*99.92*	*29865*
Severe acute respiratory syndrome coronavirus 2 isolate SARS-CoV-2/human/IND/SRHU_54/2021	*54189*	*54189*	*100%*	*99.92*	*29903*

Severe acute respiratory syndrome coronavirus 2 isolate SARS-CoV-2/human/USA/CMI-IGM-004/2021	*54187*	*54187*	*100%*	*99.91*	*29803*
Severe acute respiratory syndrome coronavirus 2 isolate SARS-CoV-2/human/USA/FL-BPHL-3645/2020	*54185*	*54185*	*100%*	*99.91*	*29714*
